# Endometrial receptivity change: ultrasound evaluation on ovulation day and transplantation day during the natural frozen embryo transfer cycle

**DOI:** 10.3389/fendo.2023.1118044

**Published:** 2023-09-26

**Authors:** Xihong Li, Yangqin Peng, Yuyao Mao, Yuan Li, Fei Gong, Yan Ouyang

**Affiliations:** ^1^ Department of Imaging, Reproductive and Genetic Hospital of CITIC-Xiangya, Changsha, China; ^2^ Department of Imaging, Clinical Research Centre For Reproduction and Genetics in Hunan Province, Changsha, China; ^3^ Department of Scientific Research, Reproductive and Genetic Hospital of CITIC-Xiangya, Changsha, China; ^4^ Department of Scientific Research, Clinical Research Centre For Reproduction and Genetics in Hunan Province, Changsha, China; ^5^ Reproductive Medicine Center, Reproductive and Genetic Hospital of CITIC-Xiangya, Changsha, China; ^6^ Reproductive Medicine Center, Clinical Research Centre For Reproduction and Genetics in Hunan Province, Changsha, China

**Keywords:** endometrial receptivity, *in vitro* fertilization, frozen-thawed embryo transfer, natural cycle, ultrasound evaluation, ovulation day, transplantation day

## Abstract

**Objective:**

To obtain quantitative and comprehensive results of the changes in comprehensive ER indicators from ovulation day to transplantation day by ultrasonography during the natural frozen-thawed embryo transfer cycle (FET).

**Methods:**

This is a prospective analysis of 230 infertile women undergoing their first FET cycles from April 2019 to July 2021. To evaluate ER, ultrasound scans were performed on the days of ovulation and embryo transfer for all included patients. All included patients were divided into a pregnancy group and a nonpregnancy group according to whether clinical pregnancy was achieved. The ER changes from ovulation day to transplantation day in the overall study population (n=230), pregnancy group (n=158) and nonpregnancy group (n=72) were analyzed.

**Results:**

In the overall population, type C was predominant on ovulation day, but type B was the most common on transplantation day (P<0.001). From ovulation day to transplantation day, endometrial thickness was significantly increased (11.26 ± 2.14 vs. 11.89 ± 2.08 mm, P<0.001), but endometrial volume (4.26 ± 1.75 vs. 4.03 ± 1.62 ml, P<0.001), endometrial VI (1.34 ± 1.64 vs. 0.95 ± 1.99, P<0.001), VFI (0.47 ± 0.72 vs. 0.40 ± 1.03, P<0.001), subendometrial VI (5.04 ± 3.89 vs. 3.29 ± 2.92, P<0.001), FI (34.07 ± 4.61 vs. 33.41 ± 5.30, p=0.004), VFI (2.07 ± 2.65 vs. 1.19 ± 1.19, P<0.001) and frequency of endometrial peristalsis (2.90 ± 1.44 vs. 1.40 ± 1.41, P<0.001) were significantly decreased. In the pregnancy group, the changes in all ultrasound parameters were in the same direction as those in the overall population. In the nonpregnancy group, except for endometrial volume and VI, which showed no difference, other ultrasound parameters showed the same direction of change as those in the overall population. No significant difference was found in the pregnancy probability among the different absolute change groups.

**Conclusion:**

During a natural cycle, the morphology of the endometrium changes mostly from type C to type B, the endometrial thickness increases, and the volume decreases. The blood supply of the endometrium, the subendometrial 5 mm and the frequency of peristalsis decrease from ovulation day to transplantation day. Compared with the nonpregnancy group, the pregnancy group tended to have more obvious decreases in endometrial volume and blood flow perfusion. However, these endometrial changes do not mean that pregnancy is bound to occur. endometrial receptivity, *in vitro* fertilization, frozen-thawed embryo transfer, natural cycle, ultrasound evaluation, ovulation day, transplantation day

## Introduction

Embryo implantation is a complex process, and the two key factors affecting embryo implantation are embryo quality and endometrial receptivity (ER) (1). Currently, transvaginal sonography (TVS) is a routine examination method for assessing ER because of its convenience and noninvasive characteristics. Two-dimensional (2D) ultrasound can be used to assess endometrial thickness, endometrial pattern and peristalsis (2–4). However, it is difficult to obtain endometrial volume and accurate and valid measurements of blood perfusion. In contrast, three-dimensional (3D) ultrasound and power Doppler can offer comprehensive assessment of endometrial volume and endometrial and subendometrial vascularization by using 3 indices: vascularization index (VI), flow index (FI), and vascularization-flow index (VFI) (5, 6).

In previous studies, the monitoring of ER was concentrated mostly around the human chorionic gonadotrophin (hCG) trigger day in fresh cycles (7, 8) or at the end of the endometrial proliferation phase in frozen-thawed embryo transfer (FET) cycles (9, 10). The transplantation day is usually considered to be within the “window of implantation”, and therefore, it is reasonable to assume that the transplantation day is also a representative time to evaluate ER (11). We know that endometrial thickness and the endometrial pattern change throughout the menstrual cycle. However, few studies have focused on the changes in various ER indicators, and even when studies did focus on these factors, they usually addressed one or two receptivity indicators, and their conclusions were contradictory (12–15). Understanding the changes in ER and identifying the parameters that contribute to IVF success would be helpful for patient counseling, physician decision-making and clinical practice. To the best of our knowledge, no relevant studies have simultaneously analyzed the changes in multiple ER ultrasound indicators from ovulation day to transplantation day in a natural cycle.

The aim of this analysis was to obtain quantitative and comprehensive results of the changes in comprehensive ER indicators from ovulation day to transplantation day by ultrasonography. However, the physiology of endometrial development and changes during the menstrual cycle may differ under different hormone environments. Thus, in this study, we chose only patients who underwent FET by natural cycles after the first stimulated IVF treatment to reduce interference factors and ensure consistency.

## Material and methods

### Study design and participants

This prospective study of 230 infertile women undergoing FET was conducted at the Reproductive and Genetic Hospital of CITIC-Xiangya from April 2019 to July 2021. Written informed consent was obtained from all participants. This study was approved by the Ethics Committee of the Reproductive and Genetic Hospital of CITIC-Xiangya (date of approval: 11 September 2019; reference number: LL-SC-2019-023; Changsha, China).

The inclusion criteria were as follows (1): received FET after the first stimulated IVF treatment and had no history of assisted reproduction in other hospitals (2); underwent a natural FET cycle (3); age 20-35 years (4); body mass index 18-24 kg/m2; and (5) had at least 1 high-quality embryo. The exclusion criteria were as follows (1): endometriosis, adenomyosis, or adenomyoma (2); intrauterine adhesion or endometritis (3); congenital uterine malformations (4); untreated hydrosalpinx; or (5) uterine cavity effusion caused by cesarean section incision and diameter ≥2 mm.

### *In vitro* fertilization procedure

All included patients underwent controlled ovarian hyperstimulation and HCG injection. Depending on the cause of infertility, fertilization can be achieved using standard *in vitro* fertilization or intracytoplasmic sperm injection (ICSI). Frozen embryos were transferred for all included women during natural cycles. Freezing and thawing were performed with 1,2-propanediol and sucrose as cryoprotectants according to the recommendations of the freezing and thawing kits (Vitrolife Sweden AB). Endometrial thickness and follicle diameter were monitored by transvaginal sonography (TVS) from the 10-12^th^ day of the menstrual cycle. Thawed cleavage-stage embryos were transferred on day 3, and blastocysts were transferred on day 5 after ovulation.

All patients received high-quality embryo transfer. The embryo morphology was scored based on the ASEBIR consensus (16). An embryo with at least 7 blastomeres, fragmentation ≤ 10%, uniform blastomeres, no vacuoles, normal zona pellucida and blastocysts with grade 3BB and above were classified as high-quality. A maximum of two embryos can be transferred.

### Ultrasound measurement

The ultrasound scans were performed on the day of ovulation and embryo transfer for all included patients to evaluate ER. All ultrasound parameters, including endometrial thickness, morphology, volume, movement and blood perfusion, were examined by a senior ultrasonic doctor (Dr. Li) using the same ultrasound instrument (GE VOLUSON E8, General Electric Tech Co., Ltd., New York, USA) equipped with a 5–9 MHz transvaginal 3D probe.

### Two-dimensional grayscale ultrasound mode

The maximum diameter of the endometrium was measured in the longitudinal plane. The morphology of the endometrium adopts Gonen classification criteria (17), in which the endometrial pattern is classified into 3 types. Type A: an entirely homogeneous, hyperechogenic endometrium by increased reflectivity and the central echogenic line is not visualized; Type B: the endometrium with the same reflectivity compared to the surrounding myometrium, with a nonprominant or absent central echogenic line; Type C: a “triple-line” endometrium, consisting of prominent outer and central hyperechogenic line and inner hypoechogenic or black regions.

The movement of the endometrium was observed and recorded within 3 minutes and divided into 5 types according to Ljland et al. (18) (1): positive wave, the peristaltic wave from the cervix to fundus (2); negative wave, the peristaltic wave from the fundus to the cervix (3); static wave, the endometrium in a static state (4); bidirectional wave, the endometrium at the fundus and the cervix contract simultaneously; and (5) random wave, irregular movement type with uncertain direction or multiple starting points.

### Two-dimensional color Doppler ultrasound examination

The endometrial blood perfusion classification was based on the Applebaum classification standard (19): I: vessels penetrating the outer hypoechogenic area surrounding the endometrium but not entering the hyperechogenic outer margin; II: vessels penetrating the hyperechogenic outer margin of the endometrium but not entering the hypoechogenic inner area; III: vessels entering the hypoechogenic inner area of the endometrium.

### Three-dimensional ultrasound examination

The ultrasound machine was switched to the 3D mode with power Doppler. The endometrial cavity was covered by the sector of interest in the longitudinal plane. The sweep angle was set to 90 degrees to ensure a sufficiently wide scanning scope. The endometrium was outlined using virtual organ computer-aided analysis (VOCAL) software, and the endometrial volume, VI, VFI and FI were obtained (20, 21).

### Outcome measures

Serum human HCG levels were measured 14 days (12 days of blastocysts) after transfer, and TVS scans were performed 4 weeks after transfer to observe the location and viability of pregnancies. The primary outcome of this study was clinical pregnancy. Ultrasound detection of an intrauterine pregnancy was considered a clinical pregnancy. All included patients were divided into a pregnancy group and a nonpregnancy group according to whether clinical pregnancy was achieved. The ER changes from the ovulation day to the transplantation day in the overall study population, pregnancy group and nonpregnant group were analyzed.

### Statistical analysis

The distribution of patient demographics was analyzed using the Kolmogorov–Smirnov test. Continuous variables are expressed as the mean ± standard deviation (SD). Categorical variables are described as the frequency and percentage. The Mann−Whitney U test or Student’s t-test was used to assess continuous variables, and the chi-square test or Fisher’s exact test was used to assess differences in categorical variables between the pregnancy group and the nonpregnancy group. Differences in continuous ultrasound indicators between the ovulation day and transplantation day were assessed by the Wilcoxon signed-rank test or paired t test, and categorical ultrasound indicators were assessed by McNemar’s chi-square test. All statistical analyses were performed using SPSS 25.0 software (IBM, Armonk, NY, United States), and a two-sided p value < 0.05 was considered statistically significant.

## Results

### Patient characteristics and ultrasound parameters

A total of 230 patients who underwent FET were included. The overall clinical pregnancy rate was 68.7% (158/230). The basic, clinical and endometrial ultrasound characteristics of the overall group, pregnancy group and nonpregnancy group are displayed in **Table 1**. There were no statistically significant differences in female age, duration of infertility, type of infertility, body mass index, cause of infertility, antral follicle count, basal hormonal levels, number of embryos transferred, or proportion of the stages of embryo transfer between the pregnancy and nonpregnancy groups (P > 0.05).

**Table 1:** Baseline and ultrasound characteristics of the overall and pregnancy and nonpregnancy groups.

Characteristic	Overall	Nonpregnancy group	Pregnancy group	p value
	Mean ± SD/n (%)	Mean ± SD/n (%)	Mean ± SD/n (%)	
Cycles	230	72(31.3%)	158(68.7%)	
Baseline characteristics				
Age (years)	30.04 ± 3.34	30.10 ± 3.35	30.02 ± 3.35	0.764
Duration of infertility (years)	3.79 ± 2.42	4.14 ± 2.88	3.63 ± 2.17	0.347
BMI (kg/m2)	21.30 ± 1.77	21.16 ± 1.82	21.36 ± 1.74	0.290
Type of infertility				0.090
Primary	102/230(44.3%)	26/72(36.1%)	76/158(48.1%)	
Secondary	128/230(55.7%)	46/72(63.9%)	82/158(51.9%)	
Cause of infertility				0.153
Unexplained	2/230(0.9%)	0/72(0%)	2/158(1.3%)	
Male factor	14/230(6.1%)	6/72(8.3%)	8/158(5.1%)	
Female factor	106/230(46.1%)	39/72(54.2%)	67/158(42.4%)	
Male and female factors	108/230(47.0%)	27/72(37.5%)	81/158(51.3%)	
AFC	22.75 ± 12.73	20.62 ± 8.86	23.73 ± 14.09	0.423
Basal hormonal levels				
FSH (mIU/ml)	5.94 ± 2.24	6.25 ± 3.29	5.79 ± 1.54	0.221
LH (mIU/ml)	3.95 ± 3.62	3.90 ± 2.64	3.98 ± 3.99	0.701
E2 (pg/ml)	48.64 ± 95.96	57.64 ± 160.48	44.57 ± 42.59	0.837
PRL (ng/ml)	20.03 ± 17.37	19.23 ± 19.26	20.41 ± 16.47	0.179
P (ng/ml)	0.76 ± 2.45	0.85 ± 2.37	0.71 ± 2.50	0.318
AMH (ng/ml)	5.44 ± 4.17	5.40 ± 3.83	5.47 ± 4.33	0.576
Number of embryos transferred				0.167
1	81/230(35.2%)	30/72(41.7%)	51/158(32.3%)	
2	149/230(64.8%)	42/72(58.3%)	107/158(67.7%)	
Stage of embryo transferred				0.902
Cleavage-stage embryo	110/230(47.8%)	34/72(47.2%)	76/158(48.1%)	
Blastocyst	120/230(52.2%)	38/72(52.8%)	82/158(51.9%)	
Ultrasound indicators on ovulation day				
Endometrial morphology classification				
Type A	12 (5.2%)	3 (4.2%)	9 (5.7%)	0.637
Type B	23 (10.0%)	9 (12.5%)	14 (8.9%)	
Type C	195 (84.8%)	60 (83.3%)	135 (85.4%)	
Endometrial blood flow classification				
I	28 (12.2%)	10 (13.9%)	18 (11.4%)	0.641
II	188 (81.7%)	59 (81.9%)	129 (81.6%)	
III	14 (6.1%)	3 (4.2%)	11 (7.0%)	
Endometrial thickness (mm)	11.26 ± 2.14	11.44 ± 2.28	11.18 ± 2.08	0.333
Endometrial volume (ml)	4.26 ± 1.75	4.17 ± 1.69	4.30 ± 1.78	0.780
Endometrial VI	1.34 ± 1.64	1.50 ± 1.92	1.26 ± 1.50	0.403
Endometrial FI	29.40 ± 7.11	30.17 ± 7.01	29.05 ± 7.15	0.678
Endometrial VFI	0.47 ± 0.72	0.59 ± 0.96	0.41 ± 0.58	0.265
Sub-Endometrial volume (ml)	11.59 ± 2.67	11.49 ± 2.46	11.63 ± 2.77	0.720
Sub-Endometrial VI	5.04 ± 3.89	5.73 ± 4.07	4.72 ± 3.77	0.068
Sub-Endometrial FI	34.07 ± 4.61	34.53 ± 4.98	33.86 ± 4.43	0.479
Sub-Endometrial VFI	2.07 ± 2.65	2.14 ± 1.73	2.03 ± 2.98	0.082
Frequency of endometrial peristalsis (times/min)	2.90 ± 1.44	2.86 ± 1.56	2.92 ± 1.38	0.393
Ultrasound indicators on ET day				
Endometrial morphology classification				
Type A	21 (9.1%)	7 (9.7%)	14 (8.9%)	0.408
Type B	200 (87.0%)	64 (88.9%)	136 (86.1%)	
Type C	9 (3.9%)	1 (1.4%)	8 (5.1%)	
Endometrial blood flow classification				
I	42 (18.3%)	13 (18.1%)	29 (18.4%)	0.725
II	181 (78.7%)	58 (80.6%)	123 (77.8%)	
III	7 (3.0%)	1 (1.4%)	6 (3.8%)	
Endometrial thickness (mm)	11.89 ± 2.21	11.83 ± 2.47	11.91 ± 2.08	0.980
Endometrial volume (ml)	4.03 ± 1.62	3.95 ± 1.53	4.07 ± 1.66	0.853
Endometrial VI	0.95 ± 1.99	1.05 ± 2.13	0.91 ± 1.93	0.786
Endometrial FI	28.21 ± 8.3	27.63 ± 9.34	28.48 ± 7.81	0.191
Endometrial VFI	0.40 ± 1.03	0.42 ± 1.06	0.38 ± 1.02	0.920
Sub-Endometrial volume (ml)	11.42 ± 2.46	11.38 ± 2.49	11.43 ± 2.45	0.860
Sub-Endometrial VI	3.29 ± 2.92	3.49 ± 3.17	3.20 ± 2.81	0.590
Sub-Endometrial FI	33.41 ± 5.30	33.39 ± 6.47	33.42 ± 4.70	0.934
Sub-Endometrial VFI	1.19 ± 1.19	1.29 ± 1.35	1.15 ± 1.11	0.703
Frequency of endometrial peristalsis (times/min)	1.40 ± 1.41	1.05 ± 1.19	1.56 ± 1.47	0.019

SD, standard deviation; BMI, body mass index; AFC, antral follicle count; FSH, follicle-stimulating hormone; LH, luteinizing hormone; E2, estradiol; PRL, prolactin; P, progesterone; AMH, anti-Müllerian hormone; VI, vascularization index; FI, flow index; VFI, vascularization-flow index.

In the comparison of the ultrasound parameters of ER between the two groups, there were no statistically significant differences in any ultrasound parameters on the ovulation day, and only the frequency of endometrial peristalsis in the pregnancy group was significantly higher than that in the nonpregnancy group on the transplantation day (1.56 ± 1.47 vs. 1.05 ± 1.19, P=0.019).

### Changes in ultrasound indicators between ovulation day and transplantation day in the overall population and two groups

Except for endometrial blood flow classification, endometrial FI and subendometrial volume, other ultrasound indicators were significantly changed between ovulation day and transplantation day for the overall population (**Table 2**). Type C endometrial morphology classification was predominant on the ovulation day, but type B was the most common on the transplantation day (P<0.001). Endometrial thickness was significantly increased from ovulation day to transplantation day (11.26 ± 2.14 mm vs. 11.89 ± 2.08 mm, P<0.001), but endometrial volume, endometrial VI, VFI, subendometrial 5-mm VI, FI, VFI and frequency of endometrial peristalsis were significantly decreased on the transplantation day (p < 0.05).

**Table 2:** Changes in ultrasound indicators between ovulation day and ET day.

	Ovulation day			ET day			p value		
	1	2	3	4	5	6	1 vs. 4	2 vs. 5	3 vs. 6
	Overall	Nonpregnancy group	Pregnancy group	Overall	Nonpregnancy group	Pregnancy group			
Cycles	230	72	158	230	72	158			
Endometrial morphology classification									
Type A	12 (5.2%)	3 (4.2%)	9 (5.7%)	21 (9.1%)	7 (9.7%)	14 (8.9%)	<0.001	<0.001	<0.001
Type B	23 (10.0%)	9 (12.5%)	14 (8.9%)	200 (87.0%)	64 (88.9%)	136 (86.1%)			
Type C	195 (84.8%)	60 (83.3%)	135 (85.4%)	9 (3.9%)	1 (1.4%)	8 (5.1%)			
Endometrial blood flow classification									
I	28 (12.2%)	10 (13.9%)	18 (11.4%)	42 (18.3%)	13 (18.1%)	29 (18.4%)	0.068	0.403	0.140
II	188 (81.7%)	59 (81.9%)	129 (81.6%)	181(78.7%)	58 (80.6%)	123 (77.8%)			
III	14 (6.1%)	3 (4.2%)	11 (7.0%)	7 (3.0%)	1 (1.4%)	6 (3.8%)			
Endometrial thickness (mm)	11.26 ± 2.14	11.44 ± 2.28	11.18 ± 2.08	11.89 ± 2.21	11.83 ± 2.47	11.91 ± 2.08	<0.001	0.042	<0.001
Endometrial volume (ml)	4.26 ± 1.75	4.17 ± 1.69	4.30 ± 1.78	4.03 ± 1.62	3.95 ± 1.53	4.07 ± 1.66	<0.001	0.055	<0.001
Endometrial VI	1.34 ± 1.64	1.50 ± 1.92	1.26 ± 1.50	0.95 ± 1.99	1.05 ± 2.13	0.91 ± 1.93	<0.001	0.077	<0.001
Endometrial FI	29.40 ± 7.11	30.17 ± 7.01	29.05 ± 7.15	28.21 ± 8.30	27.63 ± 9.34	28.48 ± 7.81	0.064	0.077	0.338
Endometrial VFI	0.47 ± 0.72	0.59 ± 0.96	0.41 ± 0.58	0.40 ± 1.03	0.42 ± 1.06	0.38 ± 1.02	<0.001	0.045	0.001
Sub-Endometrial volume (ml)	11.59 ± 2.67	11.49 ± 2.46	11.63 ± 2.77	11.42 ± 2.46	11.38 ± 2.49	11.43 ± 2.45	0.210	0.906	0.176
Sub-Endometrial VI	5.04 ± 3.89	5.73 ± 4.07	4.72 ± 3.77	3.29 ± 2.92	3.49 ± 3.17	3.20 ± 2.81	<0.001	<0.001	<0.001
Sub-Endometrial FI	34.07 ± 4.61	34.53 ± 4.98	33.86 ± 4.43	33.41 ± 5.30	33.39 ± 6.47	33.42 ± 4.70	0.004	0.045	0.038
Sub-Endometrial VFI	2.07 ± 2.65	2.14 ± 1.73	2.03 ± 2.98	1.19 ± 1.19	1.29 ± 1.35	1.15 ± 1.11	<0.001	0.001	<0.001
Frequency of endometrial peristalsis (times/min)	2.90 ± 1.44	2.86 ± 1.56	2.92 ± 1.38	1.40 ± 1.41	1.05 ± 1.19	1.56 ± 1.47	<0.001	<0.001	<0.001

ET, embryo transfer; VI, vascularization index; FI, flow index; VFI, vascularization-flow index.

The changes in all ultrasound parameters from the day of ovulation to the day of transplantation in the pregnancy group were in the same direction as those in the overall population. For the nonpregnancy group, except for endometrial volume and VI, which showed no difference, other ultrasound parameters also showed the same change direction from the day of ovulation to the day of transplantation as those in the overall population.

We further subtracted the absolute values of each ultrasound index on the ovulation day from the absolute values on the transplantation day. According to the absolute change equal to 0, greater than 0 or less than 0, the patients were classified into the no change group, increased group and decreased group. However, no significant difference was found in the pregnancy probability among the different absolute change groups (**Table 3**).

**Table 3:** Comparison of pregnancy probability in different absolute change groups of ultrasound indicators.

	No change	Increased	Decreased	p value
Endometrial thickness (mm)	3/5(60.0%)	106/150(70.7%)	49/75(65.3%)	0.558
Endometrial volume (ml)	2/2(100%)	65/95(68.4%)	91/133(68.4%)	0.784
Endometrial VI	1/1(100%)	54/82(65.9%)	103/147(70.1%)	0.622
Endometrial FI	1/1(100%)	72/100 (72.0%)	85/129(65.9%)	0.270
Endometrial VFI	3/3(100%)	57/84(67.9%)	98/143(68.5%)	0.746
Sub-Endometrial volume (ml)	–	70/105(66.7%)	88/125(70.4%)	0.543
Sub-Endometrial VI	1/1(100%)	48/68(70.6%)	109/161(67.7%)	0.563
Sub-Endometrial FI	1/1(100%)	27/65(70.7%)	45/92(67.2%)	0.491
Sub-Endometrial VFI	1/1(100%)	21/45(68.2%)	51/112(68.7%)	0.930
Frequency of endometrial peristalsis (times/min)	16/24(66.7%)	27/35(77.1%)	115/171(67.3%)	0.503

VI, vascularization index; FI, flow index; VFI, vascularization-flow index.

## Discussion

In this study, we conducted an ultrasound evaluation of the ER change from the ovulation day to the transplantation day during a natural cycle of FET. The results showed that the morphology of the endometrium changed mostly from type C to type B and that the endometrial thickness increased and the volume decreased. The blood supply of both the endometrium (VI, VFI), the subendometrial 5 mm (VI, FI, VFI) and the frequency of peristalsis decreased. In the pregnancy group, the change trend of ER was consistent with that of the overall population. Compared with those in the nonpregnancy group, endometrial volume and blood flow perfusion tended to decrease more obviously in the pregnancy group. However, the endometrium of the pregnancy group tended to have some changes, though this did not mean that pregnancy was bound to occur.

### Strengths and limitations

Our present work has some notable strengths. First, this is the first prospective cohort study to simultaneously analyze the changes in multiple ER ultrasound indicators from ovulation day to transplantation day in a natural cycle. Second, all ultrasound measurements were carried out by a senior ultrasound doctor using the same ultrasound instrument according to the standardization requirements, ensuring the consistency and standardization of measurements. Third, we grouped and analyzed the changes in ER in the pregnancy group, nonpregnancy group and overall population. Fourth, this study covers a wide range of indicators, including 2D, 3D, color Doppler and power Doppler parameters. Additionally, we chose only patients who underwent FET by natural cycles after the first stimulated IVF treatment to eliminate interference as much as possible and ensure patient consistency.

Our study also has some limitations. First, this study addressed only the natural cycle of the infertile population, and the results may not be applicable to fresh embryos and noninfertile populations. Second, we addressed only clinical pregnancy here but did not track live births. Third, the sample size was limited due to the strict inclusion and exclusion criteria. Fourth, the group with no changes in multiple indicators obtained a clinical pregnancy rate of 100%, and there were no differences in pregnancy probability among the different absolute change groups, which may be related to the small sample size. The reliability of these results needs to be further verified by larger samples. Moreover, we did not further study the difference in ER between cleavage-stage embryo and blastocyst transfer due to the sample size.

### Interpretations

ER is a period of endometrial maturation during which the trophectoderm of the blastocyst can attach to the endometrial epithelial cells and subsequently proceed to invade the endometrial stroma and vasculature (22). Diagnosis of ER has posed a challenge, and thus far, there is no worldwide accepted standardized evaluation process. Ultrasound has received increasing attention in infertility treatment, and the use of high-resolution transvaginal probes makes it possible to follow endometrial changes throughout the cycle.

The thickness, morphology and peristalsis of the endometrium can be observed under 2D grayscale ultrasound. Endometrial thickness is the most frequently observed indicator of ER. Some studies believe that endometrial compaction is beneficial to embryo implantation (11, 23), but some studies hold objections to this (13, 24). In our study, regardless of pregnancy, the endometrial thickness on the transplantation day was significantly higher than that on the ovulation day. Different research results may be related to diverse research populations and different pregnancy assistance programs. In addition, endometrial morphology changed regardless of pregnancy, and most cases were type C on ovulation day and type B on transplantation day. The endometrium changed from a triple line pattern to a nontriple line pattern in the natural cycle, but there was no significant relationship between endometrial morphology on the transplantation day and pregnancy outcome, which was also consistent with previous studies (25, 26).

In our study, endometrial peristalsis tended to be stable from ovulation to the transplantation day regardless of pregnancy, which was also consistent with research on the natural menstrual cycle, and the relative immobility of the endometrium was conducive to embryo implantation (27). It is worth noting that the frequency of endometrial peristalsis in the pregnancy group on the transplantation day is higher than that of the nonpregnancy group, which might suggest that the endometrium on the transplantation day tends to be calm but still needs some peristalsis to enable embryo implantation.

Color Doppler can be used to observe endometrial blood flow. In this study, type II blood flow was dominant on both the ovulation day and transplantation day regardless of pregnancy. However, under 3D and power Doppler, endometrial and subendometrial blood perfusion showed a decreasing trend from the ovulation day to the transplantation day, indicating that 3D and power Doppler were more sensitive to blood flow assessment than color Doppler. In addition, the changes in endometrial blood perfusion in the nonpregnancy group were less obvious than those in the pregnancy group, which means that whether the decreased endometrial blood perfusion on the transplantation day may be related to embryo implantation needs further verification in the future.

In addition, 3D ultrasound can also be used to measure the endometrial volume, which is one of the most commonly used ER indicators. We found that although the thickness of the endometrium increased, the volume of the endometrium decreased, and this trend was more significant in the pregnancy group. A recent study on fresh embryo transfer also found that compared with that on the hCG day, the endometrial thickness was significantly higher the endometrial volume significantly smaller on the transplantation day (24). Thus, endometrial volume measured by 3D ultrasound might better reflect the compact state of the endometrium, as it measures the whole endometrium rather than a selected plane.

## Conclusion

During a natural cycle, the morphology of the endometrium changed mostly from type C to type B, the endometrial thickness increased, and the volume decreased. The blood supply and the frequency of peristalsis decreased from ovulation day to transplantation day. Compared with those in the nonpregnancy group, endometrial volume and blood flow perfusion tended to decrease more obviously in the pregnancy group, but this did not mean that pregnancy was bound to occur.

## Data availability statement

The original contributions presented in the study are included in the article/supplementary material. Further inquiries can be directed to the corresponding author.

## Ethics statement

The studies involving human participants were reviewed and approved by the Ethics Committee of the Reproductive and Genetic Hospital of CITIC-Xiangya. The patients/participants provided their written informed consent to participate in this study.

## Author contributions

XL and YP contributed equally to this work and are cofirst authors of the article. XL and YO designed the study and acquired funding. Material preparation and data collection were performed by YL, FG, and YM. YP and YM performed the data analysis. XL, YP and YO wrote the manuscript. FG, YM and YL provided feedback. All authors contributed to the article and approved the submitted version.
